# Prevention and management of coronavirus disease 2019 (COVID-19) in prison: Feedback from an experience in a French remand center

**DOI:** 10.1017/ice.2020.1392

**Published:** 2020-12-15

**Authors:** Jérémy Picard, Gwenael Cornec, Elisabeth Gravrand, Séverine Ansart, Eric Stindel, Raoul Baron, Philippe Saliou

**Affiliations:** 1 Infection CONTROL UNIT, Brest University Hospital Centre, Brest, France; 2 Unité de consultation et soins ambulatoires, Brest University Hospital Centre, Brest, France; 3 Tropical and Infection Diseases Unit, Brest University Hospital Centre, Brest, France; 4 Laboratory of Medical Information Processing, LaTIM–UMR 1101, INSERM, Université de Bretagne Occidentale, Brest, France; 5 Orthopedics and Trauma Surgery Unit, Brest University Hospital Centre, Brest, France; 6 Univ Brest, Inserm, EFS, UMR1078, GGB, F-29200, Brest, France

**Fig. 1.**
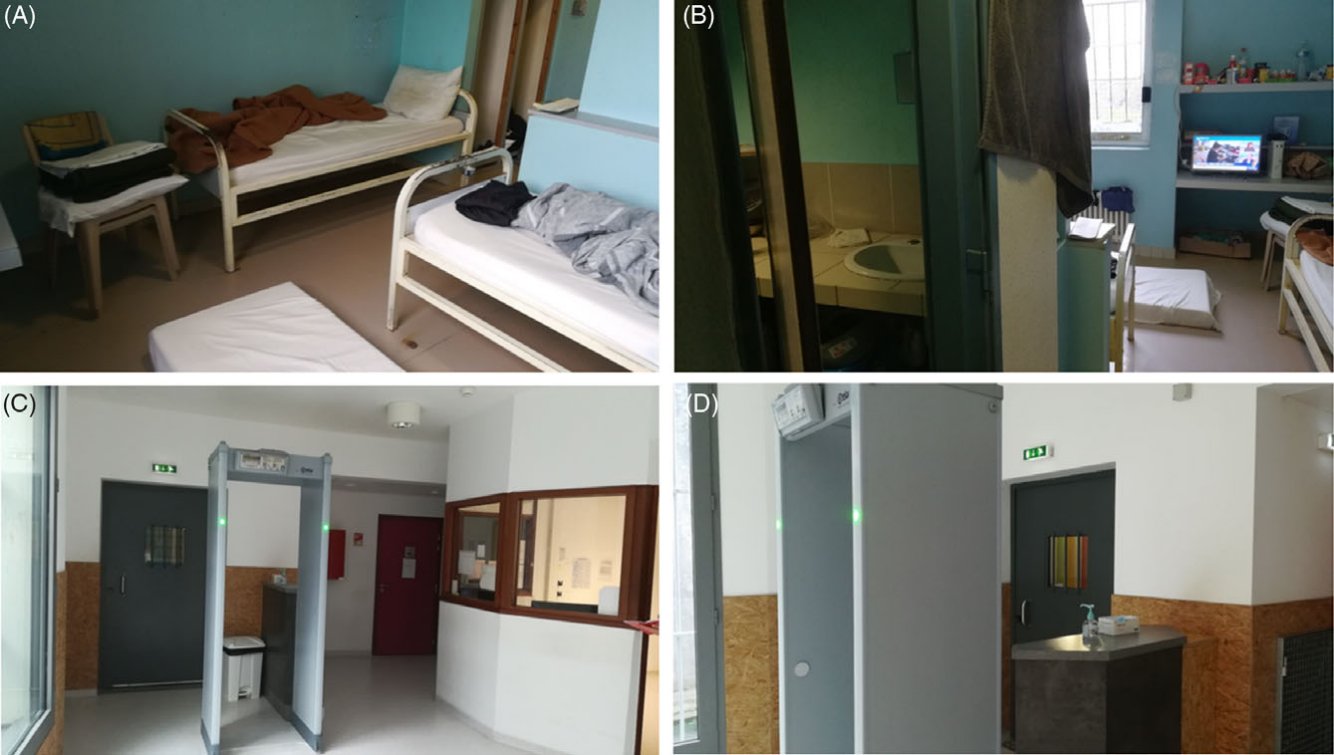
Illustration of the promiscuity of cells and prison overcrowding. (A) A mattress is placed on the floor in a cell designed to accommodate 2 inmates. (B) The bathroom is small, cluttered and difficult to clean. (C) The health unit was reorganized to avoid COVID-19 transmission. (D) Inmates disinfect their hands and put on a mask as soon as they enter the health unit.

*To the Editor—*The prisons are at high risk of coronavirus disease 2019 (COVID-19) epidemics because they concentrate a disadvantaged population within a significantly small proximity (Fig. 1).^1,2^ Faced with the spread of severe acute respiratory coronavirus virus 2 (SARS-CoV-2), French prison authorities and international learned societies have issued recommendations to organize prison health care units.^3–6^ These structures located within the prisons were created in France in 1994 and operate thanks to the university hospital center. We reorganized the prison in Brest, France, to respond to the COVID-19 pandemic.

First, we implemented the following measures: any detained person arriving in prison must disinfect their hands with alcohol-based hand sanitizer (ABHS), must wear a surgical mask, must take his temperature, and must declare any clinical signs. We also implemented numerous additional measures. Detainees are isolated in a cell for 14 days before joining the detention quarters. Supervisors and detainees routinely wear surgical masks during close physical contact. Walks are authorized in compliance with social distancing measures: wearing a surgical mask, respecting the distances between inmates, disinfecting the hands with ABHS.

At the end of the confinement, personal linen is placed in water-soluble bags collected by the supervisors. The laundry is washed at 60°C for 30 minutes in a dedicated machine. After 3 hours of ventilation, the cell is disinfected with bleach diluted with 0.5% active chlorine. The prisoner is then relocated in his neighborhood of origin.

Prisoners suspected of COVID-19 are screened in the health unit by nasopharyngeal swab. The prisoner and their codetainees are confined to their cells pending the results. If the test is positive for COVID-19, the prisoner remains confined for 14 days. He is allowed to go for a walk alone, equipped with a surgical mask. He must not have direct contact with other prisoners. Inmates who have shared the same cell are also confined alone for 14 days.

All medical, paramedical, and penitentiary personnel must wear surgical masks in the care unit. Prisoners with signs suggestive of COVID-19 are isolated from other patients in a specific room for screening. The consultation rooms are disinfected after each passage of inmates and are ventilated for 3 hours in the event of suspicion of COVID-19.

The management of COVID-19 in prison must include systematic screening of new arrivals and suspected persons, cohorting and social distancing, work stoppage for professionals contaminated, and training of professionals and inmates regarding hygiene precautions.^7^ During the first epidemic wave, the French penitentiary authorities suspended visits to prisoners. Thanks to these measures, the Brest detention center has not yet experienced a COVID-19 epidemic.

The French authorities have released detainees who could benefit from a reduction in sentences or who were reaching the end of their sentence. The COVID-19 epidemic also led to the termination of judicial proceedings, which has limited the number of admissions to prison. The number of prisoners in France fell from 72,575 on March 15, 2020, to 58,926 on May 24, 2020.^8^ The prison occupancy rate has therefore fallen sharply, falling below the 100% occupancy mark. Thus, the men’s detention area in Brest, which had an occupancy rate of 185.8% on January 1, 2020, has seen its occupancy rate fall below 100%.^9^

It is essential that the prison authorities of countries organize the fight against COVID-19 thanks to the resources available in hospitals such as hygiene teams.^10^ All of these measures must be guided by recommendations published by learned societies, and financial additional funds should be granted to prisons.
